# Photobiomodulation Therapy in Head and Neck Cancer Patients Undergoing Chemoradiotherapy: Role in Toxicities Prevention

**DOI:** 10.3390/jcm15207968

**Published:** 2026-10-15

**Authors:** Margherita Rotondi, Roger Sun, Houssam EL-Masri, Giuseppe Facondo, Andreea Cabulca, Nahla Belattar, Gabriela Dumitrescu, Eric Deutsch, Camelia Billard-Sandu

**Affiliations:** 1 Department of Radiation Oncology, Institute Gustave Roussy, 94805 Villejuif, France; roger.sun@gustaveroussy.fr (R.S.); houssam.el-masri@gustaveroussy.fr (H.E.-M.); camelia.billard@gustaveroussy.fr (C.B.-S.); 2 INSERM, Radiothérapie Molèculaire et Innovation Thérapeutique, 94805 Villejuif, France; 3 Department of Radiation Oncology, Hospital Santa Maria della Misericordia, 33100 Udine, Italy; facondogiuseppe@gmail.com

**Keywords:** photobiomodulation, low level laser therapy, head and neck cancer, chemotherapy, radiotherapy, toxicities, orofacial complications, supportive care

## Abstract

**Background:** Chemoradiotherapy (CRT) for head and neck cancer (HNC) is frequently associated with severe acute toxicities that compromise treatment adherence and quality of life. This study evaluated the effectiveness of photobiomodulation (PBM) in reducing CRT-induced toxicities in a real-world cohort. **Methods:** Between May 2021 and July 2024, we retrospectively analyzed 117 HNC patients treated with definitive or adjuvant CRT and concomitant PBM: a non-invasive application of red or near-infrared light to cutaneous and mucosal surfaces to modulate inflammatory responses, using 620/820 nm or 650 nm devices, with irradiation times of 2–12 min and energy densities of 3–12 J/cm^2^ according to the treatment protocol. Toxicities were graded according to CTCAE v 5.0 at baseline, during treatment, at treatment completion, and throughout follow-up. Associations with toxicity were assessed with logistic regression for repeated measurements (generalized estimating equations). Patients were stratified according to PBM exposure (<10 vs. ≥10 sessions). **Results:** Median follow-up was 16 months. At treatment completion, grade 2–3 oral mucositis and radiodermatitis occurred in 38.5% and 25.6% of patients, respectively, both lower than rates reported in historical cohorts without PBM. Patients receiving ≥10 PBM sessions (72 pts) experienced significantly lower rates of grade 2–3 odynophagia at treatment completion (16.7% vs. 42.2%, *p* = 0.005) and persistent dysphagia at the last follow-up (12.5% vs. 33.3%, *p* = 0.009). PBM exposure was not associated with significant differences in weight loss, progression-free survival, or overall survival. **Conclusions:** This study provides real-world data on toxicity outcomes among HNC patients receiving PBM, with lower rates of selected toxicities observed among patients receiving ≥10 PBM sessions. Although the retrospective design and lack of a control group limit causal inference, these findings provide a strong rationale for prospective randomized trials to define the optimal PBM schedule and validate its clinical impact.

## 1. Introduction

Head and neck cancers (HNC) comprise a heterogeneous group of tumors requiring multidisciplinary approach. Adjuvant chemoradiotherapy (CRT) and definitive CRT, are cornerstone strategies in the management of HNC patients [1,2]. Although these strategies enhance oncologic outcomes, they also induce toxicities that can affect quality of life (QoL) potentially leading to treatment delays or interruptions, thus compromising treatment efficacy. Radiotherapy-induced toxicities such as radiodermatitis, oral mucositis, dysphagia, xerostomia, oral pain, and dysgeusia contribute to malnutrition and weight loss, further exacerbating patient vulnerability during treatment [3]. To mitigate these adverse effects, the European Society for Medical Oncology (ESMO) recommends photobiomodulation (PBM) therapy—formerly known as low-level laser therapy— a non-invasive application of red or near-infrared light, delivered via LASER (light amplification by stimulated emission of radiation) or LED (light-emitting diode), to cutaneous and mucosal surfaces. This light energy is converted into metabolic energy at the cellular level, promoting tissue repair and modulating inflammatory responses. Photobiomodulation has been shown to stimulate the synthesis of adhesion molecules, enhance leukocyte migration to sites of inflammation, and support adaptive immune responses during wound healing. Additionally, it facilitates collagen fiber alignment, contributing to epidermal regeneration and scar formation [4,5].

A recent meta-analysis of eight clinical trials involving 526 patients demonstrated that PBM significantly reduces the incidence of grade 2–3 radiodermatitis in patients with breast and head and neck cancers [6]. Since 2018, the National Institute for Health and Care Excellence (NICE) has recommended PBM for the prevention of oral mucositis in HNC patients undergoing CRT [7]. Similarly, the Mucositis Study Group of the Multinational Association of Supportive Care in Cancer/International Society of Oral Oncology (MASCC/ISOO) has endorsed PBM for managing acute radiodermatitis in breast cancer and is increasingly recognizing its potential in HNC [8,9,10]. The objective of this study is to evaluate, in a real-world cohort treated at our institution, the impact of PBM therapy on the incidence and severity of CRT-induced toxicities in patients with HNC.

## 2. Materials and Methods

### 2.1. Patients

This retrospective analysis included 117 consecutive eligible patients with HNC who received PBM therapy during CRT at Gustave Roussy Cancer Campus, Villejuif, France, between May 2021 and July 2024. All patients had histologically confirmed HNC and were treated with adjuvant or definitive CRT, with or without induction chemotherapy. All patients provided informed consent to undergo PBM therapy, initiated at the start of CRT and continued until its completion, with the aim of preventing or managing CRT-related toxicities in the head and neck region. Patients were excluded if they received radiotherapy alone, had synchronous malignancies outside the head and neck area, or initiated PBM only after completing CRT.

### 2.2. Treatment Schedule

At baseline, patients were staged using the TNM 8th edition [11], and treatment decisions were made by a multidisciplinary board. Depending on stage, tumor site, and histology, patients received either surgery with adjuvant CRT or definitive CRT, with or without induction chemotherapy. Treatment protocols were aligned with institutional standards and National Comprehensive Cancer Network (NCCN) guidelines [12]. RT was delivered using volumetric modulated arc therapy (VMAT) or Intensity-modulated radiation therapy (IMRT), administered five days a week over 6 to 7 weeks. The total dose was 64–66 Gy in 30–33 fractions for adjuvant CRT, and 70 Gy in 33–35 fractions for definitive CRT. Target volume delineation followed international consensus guidelines [13,14]. The most applied chemotherapy regimen was cisplatin 100 mg/m^2^ on days 1, 22, and 43, weekly cisplatin (40 mg/m^2^), carboplatin (AUC 4) on days 1, 22, and 43, or weekly cetuximab (400 mg/m^2^ loading dose followed by 250 mg/m^2^ weekly). Patients with cT4 and/or cN3 tumors of the nasopharynx or larynx were treated with induction chemotherapy prior to CRT.

### 2.3. PBM—Session

Photobiomodulation was administered concurrently with CRT using two CE-certified class IIa medical devices: the ATP38 (Swiss Bio Innov S.A., Moudon, Switzerland; CE certificate no. 31566, rev. 5, dated 7 July 2022) and the CareMin 650 (NeoMedlight, Villeurbanne, France; CE certificate no. 31924, rev. 5, dated 28 February 2020) (Table 1). 

Extra-oral PBM was delivered using the ATP38 LED panel device, positioned approximately 4 cm from the anterior cervical skin. Intra-oral PBM was administered using CareMinOral LED pads (NeoMedlight, Villeurbanne, France) placed directly on the oral mucosa. In routine practice, PBM sessions lasted 15–20 min. The protocol was individualized and adjusted each session to optimize efficacy and patient comfort.

### 2.4. Response and Toxicity Assessment

Acute toxicity was evaluated at four key timepoints: the first session of PBM therapy (T0), midpoint of PBM sessions (T1), completion of PBM (T2), and at the first follow-up visit, three months after the end of chemoradiotherapy (T3). Late toxicity was assessed at the last clinical follow-up (T4), including osteoradionecrosis, radiation-induced thyroiditis and ototoxicity. All toxicities were scored according to Common Terminology Criteria for Adverse Events version 5.0 (CTCAE v 5.0) [15]. Nutritional status was assessed by recording weight at the start and end of CRT and documenting enteral nutrition use. Treatment response was evaluated during follow-up through physical exam, fibrolaryngoscopy, and imaging: magnetic resonance imaging, computed tomography, and/or positron emission tomography with fluorodeoxyglucose according to national guidelines.

## 3. Statistical Analysis

Comparisons between variables were performed using the Wilcoxon signed-rank test or Kruskal–Wallis test for continuous variables, and Fisher test or χ^2^ test for categorical variables, as appropriate. Subgroup analyses based on the number of PBM sessions and the timing of PBM initiation were performed by dichotomizing each variable according to its median value. To account for repeated toxicity assessments within patients, each toxicity was analyzed as a binary outcome (any grade, grade 1–3 vs. grade 0; and clinically relevant, grade 2–3 vs. grade 0–1) using logistic regression fitted with generalized estimating equations (GEE; logit link, exchangeable working correlation, robust sandwich standard errors). Predictors were time point (T0–T4, categorical), number of PBM sessions (<10 vs. ≥10) and timing of PBM initiation (early, <7 days vs. late, ≥7 days after RT start), entered simultaneously. Results are reported as odds ratios (OR) with 95% confidence intervals (CI). Time points with no events for a given toxicity were excluded from the corresponding model. As sensitivity analyses, models were refitted (i) as generalized linear mixed models with a random intercept per patient, and (ii) excluding T0, because the first PBM session occurred after the start of RT in late-starting patients. For associations previously observed at a single time point, a time-by-PBM interaction term was tested. Given the number of toxicities examined, these analyses were considered exploratory; Benjamini–Hochberg false discovery rate-adjusted *q*-values are reported in the Supplementary Materials. Models were fitted with the geepack and lme4 R packages. *p*-values of 0.05 or less were considered statistically significant. Statistical analyses were performed using R software version 4.4.3 (https://www.r-project.org/ (accessed on 5 August 2026)) [16]. Survival outcomes were calculated from the date of CRT initiation, and were computed according to the Kaplan–Meier method and Cox proportional-hazards survival estimates. Endpoints were death from any cause for overall survival (OS), and any recurrence or death for progression-free survival (PFS).

## 4. Results

### 4.1. Patient’s Characteristics

A total of 117 patients, with a median age of 59 years (IQR, 53–66 years) and an ECOG performance status of 0–1, were treated with CRT and PBM. Median follow-up was 16 months (IQR,11–21 months). Table 2 summarizes the demographic and clinical characteristics of all patients. Systemic treatment was platinum-based in the majority of cases (114 patients, 97%), while three patients received cetuximab.

### 4.2. PBM Protocols

All patients received PBM, with the number of sessions varying mainly due to logistical and staffing limitations. The median number of sequences was 10 (IQR, 8–12 sessions), with 45 patients (38.5%) receiving fewer than 10 PBM sessions and 72 (61.5%) receiving 10 or more. All patients completed radiotherapy without interruptions longer than five days.

The distribution of PBM devices (ATP38 vs. CareMin650) and treatment protocols was assessed at three key time points (T0, T1, and T2) during PBM therapy. Data were stratified according to the total number of PBM sessions (<10 vs. ≥10) and the timing of PBM initiation (early, <7 days vs. late, ≥7 days) (Supplementary Materials Table S1).

### 4.3. Toxicity Characteristics

PBM-related toxicities were assessed at five standardized timepoints relative to the start of CRT. The first PBM session (T0) occurred a median of 9 days (interquartile range [IQR] 5–17) after the initiation of radiotherapy in patients who received <10 PBM sessions, compared to a median of 6 days (IQR 2–8.3) in those who received ≥10, with a statistically significant difference (*p* = 0.004). The mid-point of PBM treatment (T1) occurred at approximately 23 to 24 days in both groups (*p* = 0.682). The end of PBM treatment (T2) occurred significantly later in the group receiving 10 or more sessions, with a median of 46 days (IQR 43–48) versus 42 days (IQR 39–44) in the <10 sessions group (*p* < 0.001). The first follow-up (T3), conducted three months after CRT, occurred at a median of 137 days in both groups (*p* = 0.857). The last available follow-up (T4) showed a non-significant trend toward longer follow-up in the ≥10 sessions group (median: 454 days) compared to the <10 sessions group (median: 370 days). Table 3 summarizes the incidence of treatment-related toxicities across five standardized timepoints for the entire cohort.

The most frequent grade 1 toxicity was radiodermatitis at T1 (84 patients), grade 2 toxicity was mucositis at T2 (39 patients), and grade 3 toxicity was dysphagia at T2 (28 patients). No grade 4 toxicities were reported at any timepoint. A key finding in Table 3 is not only the onset of toxicities but also their recovery over time. Mucositis, dysphagia, odynophagia, and radiodermatitis worsened markedly at T1–T2, reaching their peak severity during treatment, and subsequently improved substantially at T3–T4. For example, the proportion of patients with Grade 0 mucositis decreased from 81.2% at T0 to 23.1% at T2, corresponding to the peak of mucositis severity, and subsequently increased to 97.4% at T4. In contrast, xerostomia showed a progressive and monotonic worsening over time, with the proportion of patients with Grade 0 xerostomia decreasing from 97.4% at T0 to 84.6% at T1, 77.8% at T2, and 47% at both T3 and T4. Notably, there was no evidence of recovery by T4; rather, xerostomia appeared to persist at Grade 1–2 in approximately half of the patients (Figure 1). 

Nutritional support via nasogastric tube or gastrostomy was required in 6 patients (5.1%). Toxicities according to the number of PBM sessions and the timing of PBM introduction (early vs. late onset, less or more than 7 days after CRT start) were analyzed with logistic regression accounting for repeated measurements (Table 4); descriptive data by time point are provided in Supplementary Materials Table S2. 

Time point was strongly associated with mucositis, radiodermatitis, dysphagia, odynophagia and xerostomia (global *p* < 0.001), reflecting their peak at T1–T2 and subsequent recovery, except for xerostomia, which increased progressively up to T3–T4; no significant change over time could be demonstrated for trismus.

After adjustment for time point and timing of PBM initiation, a non-significant trend towards lower odds was observed between receiving ≥10 PBM sessions and any-grade dysphagia (OR 0.64, 95% CI 0.38–1.06, *p* = 0.084).

Conversely, patients receiving ≥10 sessions had higher odds of radiodermatitis, both any grade (OR 2.31, 95% CI 1.25–4.27, *p* = 0.008) and grade ≥2 (OR 2.61, 95% CI 1.17–5.81, *p* = 0.019).

Late PBM initiation (≥7 days after RT start) was associated with higher odds of any-grade dysphagia (OR 1.72, 95% CI 1.04–2.81, *p* = 0.033), any-grade radiodermatitis (OR 1.91, 95% CI 1.04–3.49, *p* = 0.036), grade ≥2 radiodermatitis (OR 3.30, 95% CI 1.55–7.03, *p* = 0.002) and grade ≥2 xerostomia (OR 2.20, 95% CI 1.07–4.54, *p* = 0.033), with a trend for grade ≥2 odynophagia (OR 1.70, 95% CI 0.95–3.04, *p* = 0.072). Estimates were consistent in mixed-effects models; after exclusion of T0, the associations of late initiation with any-grade dysphagia and radiodermatitis were attenuated and no longer significant (Supplementary Materials Table S3).

The association between the number of PBM sessions and grade ≥2 odynophagia varied over time (interaction *p* = 0.026): ≥10 sessions were associated with lower odds of grade ≥2 odynophagia at the end of PBM (T2; OR 0.29, 95% CI 0.13–0.68, *p* = 0.005), whereas no significant association could be demonstrated at T1 or T3. Similarly, ≥10 sessions were associated with lower odds of dysphagia at the last follow-up, although the overall time-by-sessions interaction was not significant (Supplementary Materials Table S4). None of these associations remained significant after false discovery rate correction (all *q* > 0.05); these findings should therefore be regarded as hypothesis-generating.

Among late toxicities, assessed once at the last follow-up, no significant association was observed with the number of PBM sessions. Late PBM initiation was associated with lower odds of ototoxicity (OR 0.24, 95% CI 0.08–0.75, *p* = 0.014).

The analysis of weight variations across PBM treatment groups revealed no statistically significant differences between patients receiving <10 versus ≥10 PBM sessions. The median weight loss was 4.27% (IQR 0.00–9.46) in the <10 PBM group and 3.32% (IQR −0.97 to 6.37) in the ≥10 PBM group (*p* = 0.320).

### 4.4. Clinical Outcomes

Among the 117 patients included in the analysis, 22 (18.8%) experienced a PFS event, and 6 (5.1%) died during the follow-up period. The median follow-up was 16 months [IQR, 10.6–20.6].

The median OS was not reached with estimated OS rates of 97.9% (95% CI, 94.9–100) at 12 months and 88.0% (95% CI, 78.6–98.6) at 24 months. Median PFS was not reached with estimated PFS rates of 87.2% (95% CI, 80.6–94.4) at 12 months and 64.3% (95% CI, 51.2–80.7) at 24 months.

Patients who received ≥10 PBM sessions had comparable PFS (HR = 0.87, 95% CI, 0.36–2.1; *p* = 0.76) and OS (HR = 0.47, 95% CI, 0.094–2.3; *p* = 0.36) compared to those with fewer sessions (Figure 2). 

## 5. Discussion

Concurrent CRT remains the cornerstone of therapy for HNC. Its application is tailored according to tumor site, disease stage, and specific adverse risk factors. 

The toxicity burden of HNC is particularly relevant in patients receiving multimodal treatment. During CRT, acute adverse effects commonly include mucositis, odynophagia, dysphagia, dermatitis, xerostomia, taste alterations, fatigue, nausea, and nutritional deterioration [17]. These toxicities may become mutually reinforcing, as pain and mucosal injury can impair oral intake, while dysphagia, salivary dysfunction, and taste changes may further contribute to weight loss and malnutrition. Treatment-related toxicity is not, however, restricted to the acute phase. Increasing evidence indicates that HNC survivors may experience clinically meaningful late effects years after treatment completion, including xerostomia, dysphagia, soft-tissue fibrosis, voice alterations, hearing impairment, hypothyroidism, pain, trismus, and aspiration. In a recent multinational cross-sectional study of 1094 long-term HNC survivors, 68% had at least one moderate-to-severe late toxicity, with dry mouth (67%), soft-tissue fibrosis (52%), dysphagia (51%), and voice alterations (39%) among the most frequent complications. These findings highlight that treatment-related toxicity represents a persistent survivorship issue rather than a transient consequence of cancer therapy [18]. 

Photobiomodulation has emerged as a promising supportive care strategy by promoting tissue repair, modulating inflammation, and accelerating epithelial regeneration [4]. In this study, we evaluated the impact of PBM on both acute and late toxicities in a cohort of patients undergoing CRT for HNC. Although our study did not include a control group, the observed toxicity rates were in line with those reported in trials evaluating PBM, supporting the hypothesis that PBM contributes to improved tolerance of CRT. In fact, the incidence of treatment-related toxicities in our cohort appeared lower than those typically reported in historical populations treated with CRT alone. At T2, grade ≥2 mucositis was observed in 38.5% of patients (33.3% grade 2, 5.1% grade 3), which is notably lower than the 34–42% incidence of grade 3–4 mucositis reported in the pooled analysis of NRG Oncology/RTOG 0129 and 0522 trials, as well as in the meta-analysis of large clinical studies by Trotti et al. [19,20]. Similarly, grade 3 dysphagia was observed in 24% of patients in our cohort, whereas the NRG Oncology/RTOG 0129 analysis reported rates ranging from 30% to 59% at T2. Grade ≥2 radiodermatitis affected 25.6% of patients in our study, closely matching the 29% reported in the PBM arm of the DERMISHEAD randomized trial, and substantially lower than the 88% observed in its control group [21]. Importantly, no grade 4 toxicities were recorded for mucositis or dermatitis, in line with prior PBM studies [21,22].

Xerostomia also appeared reduced, with only 20.5% of patients experiencing grade ≥2 symptoms at 3 months after the end of RT, and just 6% at the final follow-up(T4). Photobiomodulation support may further enhance outcomes observed in IMRT-era trials such as PARSPORT, where Grade ≥2 xerostomia was reduced to 41% of patients at 12 months [23]. Collectively, these findings suggest a potential protective effect of PBM across multiple domains of CRT-related toxicity.

The number of PBM sessions was not associated with overall toxicity across time points, but ≥10 sessions were associated with less grade ≥2 odynophagia at the end of CRT and less dysphagia at last follow-up, suggesting a possible time-specific benefit that requires confirmation. This observation is particularly relevant given the multifactorial nature of long-term dysphagia, which often results from persistent mucosal inflammation, fibrosis, salivary gland dysfunction, and chronic pain. Dysphagia remains a major contributor to malnutrition, which affects 30–50% of patients with HNC at diagnosis and is frequently exacerbated during treatment [24].

Despite reduced treatment-related toxicities, no significant differences in weight loss were observed between subgroups. Other factors—such as nausea, vomiting, taste changes, and appetite loss—also contribute to cancer-related malnutrition. Individualized nutritional support may have helped limit weight loss across the cohort. Since all patients received PBM, the lack of a PBM-naïve control group prevents determining whether PBM directly aids weight preservation [25].

Nevertheless, the overall magnitude of weight loss (–3.4%) appears comparable to, or slightly lower than, historical CRT cohorts without PBM, where median weight loss ranged from 4.6% to 8% during treatment [26]. These findings underscore the importance of including nutritional endpoints in future trials and evaluating PBM against a control arm to better define its role in preserving functional outcomes such as swallowing, oral intake, and weight maintenance during CRT.

Unlike systemic medications used for pain management, PBM is not associated with systemic adverse effects. It is a non-invasive and well-tolerated supportive care intervention. However, PBM is time-consuming and requires the availability of healthcare personnel to perform daily, repetitive sessions. Its effectiveness may depend on the total number of sessions administered. In our study, adherence was not a concern, likely due to the integration of PBM with daily radiotherapy sessions. In contrast, very few patients returned for PBM after the completion of radiotherapy, primarily due to the long distance between their homes and the hospital. Home-use PBM devices could therefore represent a feasible maintenance strategy, potentially allowing patients to continue treatment after completion of radiotherapy while reducing the burden associated with repeated hospital visits [27].

The analysis of clinical outcomes in our cohort revealed no significant differences in PFS or OS when stratified by number of PBM sessions. Our results are consistent with current literature, which indicates that PBM does not negatively impact tumor control or survival when used adjunctively in cancer care. This reinforces the feasibility of integrating PBM into routine management for HNC patients without compromising oncologic outcomes [28].

This study has several limitations. First, its retrospective design introduces potential biases, including heterogeneity in supportive care and treatment practices. Second, the absence of a non-PBM control group prevents any causal conclusion about the effect of PBM itself. Third, the number of PBM sessions and the timing of PBM initiation were not randomly assigned. Patients who developed radiodermatitis may have been switched to curative protocols and received additional sessions, and this confounding by indication may partly explain the higher odds of radiodermatitis observed in patients receiving ≥10 sessions. Similarly, patients who started PBM later already had more mucositis and dysphagia at their first session, suggesting that PBM may have been initiated in response to emerging symptoms rather than preventively; the associations between late initiation and toxicity should therefore not be interpreted as a causal effect of timing. The suboptimal number of PBM sessions in some patients, mainly due to logistical constraints, further complicates interpretation. Fourth, given the number of toxicities and time points examined, none of the associations remained significant after correction for multiple testing, and the subgroup analyses should be regarded as exploratory and hypothesis-generating. Finally, the duration of follow-up may have been insufficient to capture late-onset toxicity fully; the results regarding late toxicities should therefore be interpreted with caution and may not yet provide a complete assessment of long-term adverse effects.

## 6. Conclusions

In conclusion, this study adds real-world evidence that PBM helps reduce CRT-related toxicities in HNC patients. Although subgroup signals regarding PBM timing and session number are exploratory, the findings support its potential clinical value. Encouraged by these preliminary findings, well-designed randomized controlled trials with standardized toxicity assessment and patient-reported outcomes are now essential to confirm efficacy and guide the integration of PBM into routine supportive care.

## Figures and Tables

**Figure 1:**
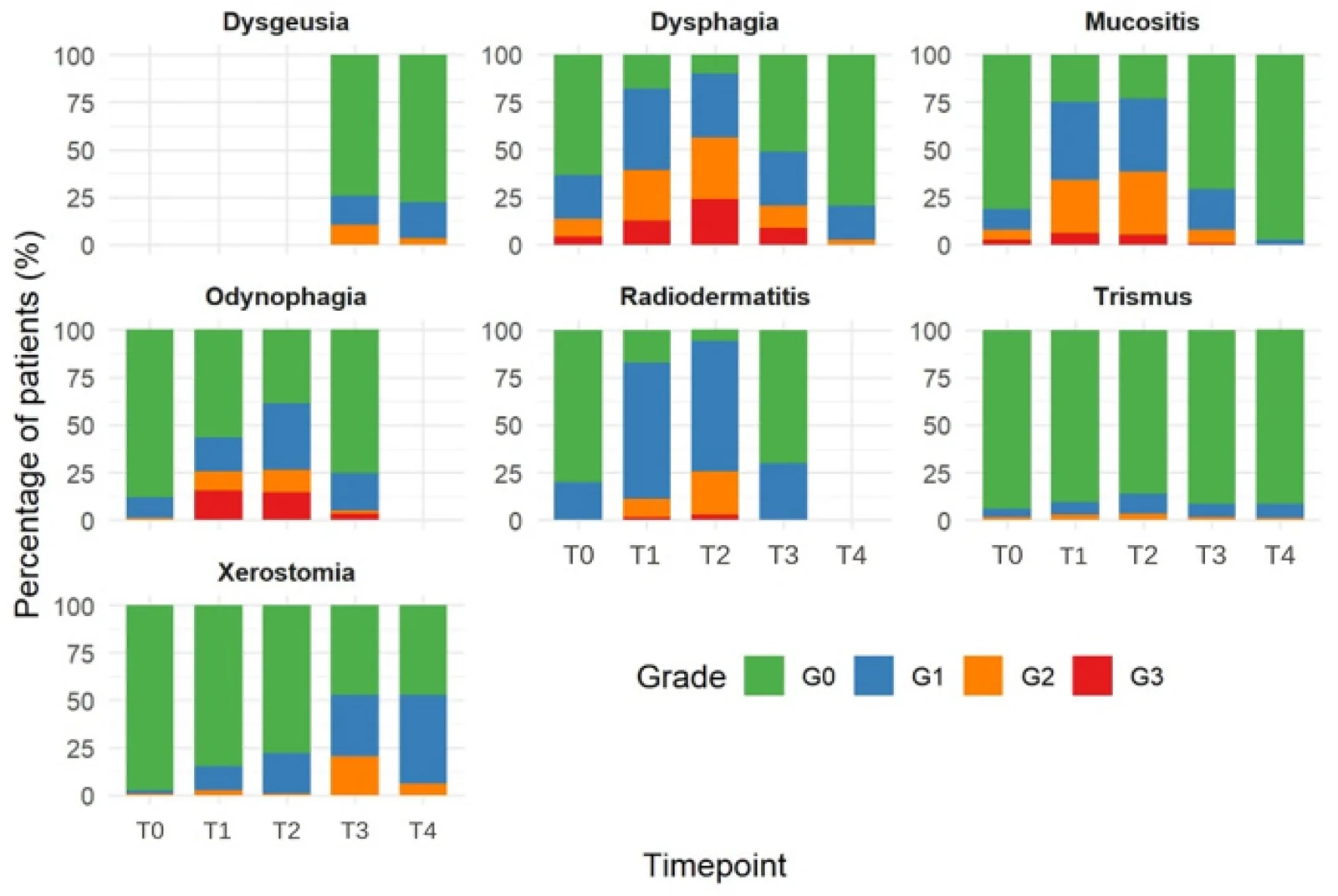
Toxicity of the overall cohort. G: toxicity grade. T0: at the beginning of PBM. T1: at the mid-way point of PBM treatment. T2: at the end of treatment. T3: at 3 months to end of treatment. T4: last follow up.

**Figure 2:**
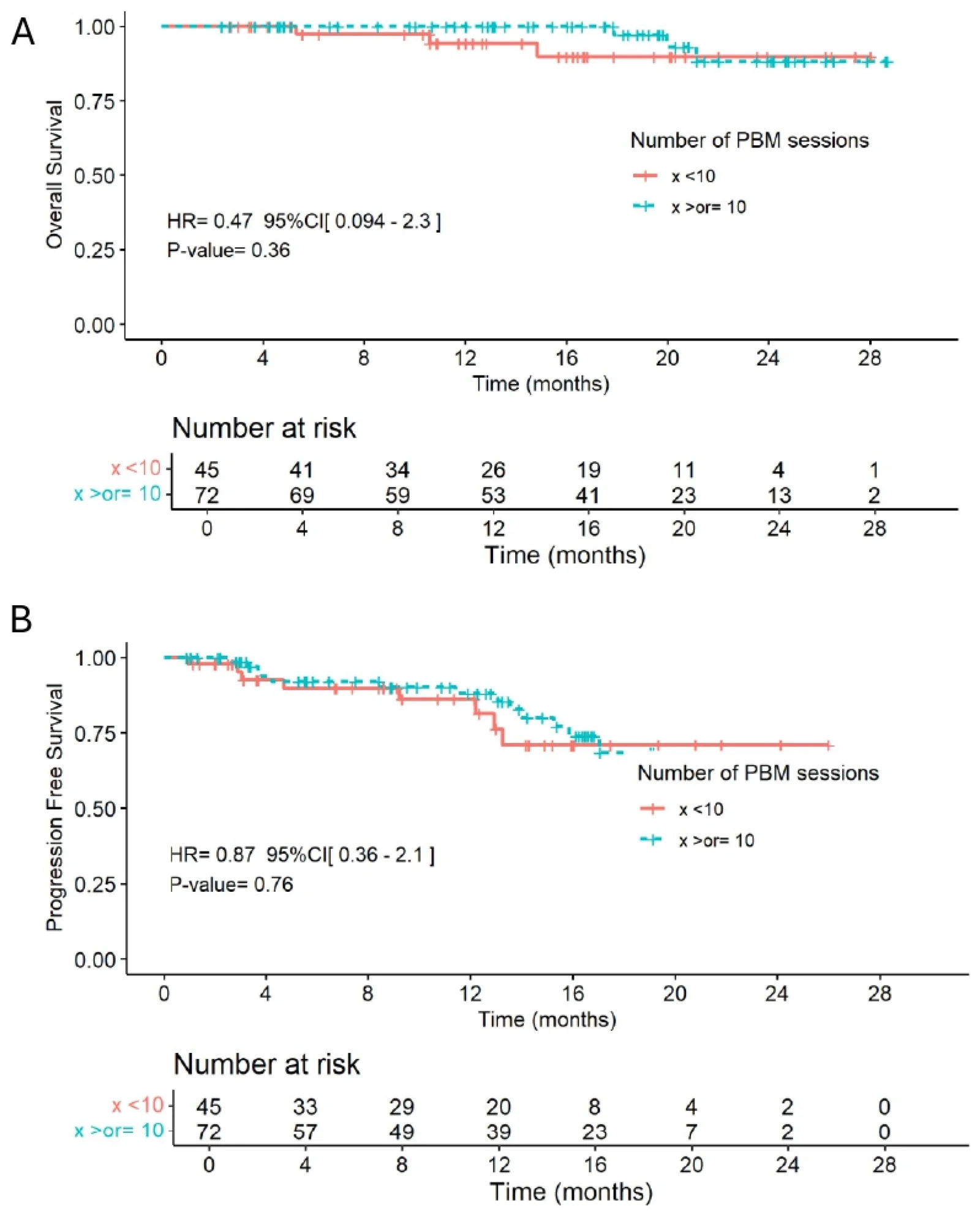
Kaplan–Meier survival curves according to the number of PBM sessions. (**A**,**B**): 10. PBM sessions (blue line) did not show significant differences in OS (HR = 0.47; 95% CI: 0.094–2.3; *p* = 0.36) or PFS (HR = 0.87; 95% CI: 0.36–2.1; *p* = 0.76) compared to those receiving <10 sessions (red line). The number of patients at risk at each time point is shown below each plot.

**Table 1:** Technical characteristics PBM devices.

Device		Wavelength (nm)	Time per Session	Energy Density (Fluency: J/cm^2^)
ATP38	Preventive radiodermatitis	620 + 820	6–8	6
	Curative radiodermatitis	620 + 820	10–12	12
	Mucositis and anti-inflammatory	620 + 820	6–8	12
CareMin650	Preventive indication	650	2	3
	Curative indication	650	3.5	6

**Table 2:** Patient characteristics.

	Level	Overall	*N* Total Sessions < 10	*N* Total Sessions ≥ 10	*p*	Early PBM (<7 Days)	Late PBM (≥7 Days)	*p*
*N* patients		117	45	72		62	55	
Age (median [IQR])		59 [53, 66]	58 [52, 66]	60 [53.75, 65.25]	0.684	59.50 [54, 65.75]	58 [52, 65]	0.658
Body mass index (%)	normal <24.9	52 (44.4)	20 (44.4)	32 (44.4)	0.93	25 (40.3)	27 (49.1)	0.523
	overweight <30	51 (43.6)	19 (42.2)	32 (44.4)		28 (45.2)	23 (41.8)	
	obesity ≥30	14 (12)	6 (13.3)	8 (11.1)		9 (14.5)	5 (9.1)	
Location (%)	oral cavity	24 (20.5)	8 (17.8)	16 (22.2)	0.799	16 (25.8)	8 (14.5)	0.28
	oropharynx	53 (45.3)	20 (44.4)	33 (45.8)		22 (35.5)	31 (56.4)	
	nasopharynx	9 (7.7)	5 (11.1)	4 (5.6)		5 (8.1)	4 (7.3)	
	hypopharynx	7 (6)	4 (8.9)	3 (4.2)		4 (6.5)	3 (5.5)	
	larynx	14 (12.0)	5 (11.1)	9 (12.5)		7 (11.3)	7 (12.7)	
	unknown primary	5 (4.3)	2 (4.4)	3 (4.2)		4 (6.5)	1 (1.8)	
	major salivary glands	5 (4.3)	1 (2.2)	4 (5.6)		4 (6.5)	1 (1.8)	
Histology (%)	squamous cell carcinoma	109 (93.2)	43 (95.6)	66 (91.7)	1.000	56 (90.3)	53 (96.4)	0.242
	adenoid cystic carcinoma	3 (2.6)	1 (2.2)	2 (2.8)		3 (4.8)	0 (0.0)	
	myoepithelial carcinoma	1 (0.9)	0 (0.0)	1 (1.4)		0 (0.0)	1 (1.8)	
	non-keratinizing undifferentiated carcinoma	4 (3.4)	1 (2.2)	3 (4.2)		3 (4.8)	1 (1.8)	
Stage (%)	I-II	28 (23.9)	6 (13.3)	22 (30.6)	0.057	11 (17.7)	17 (30.9)	0.147
	III-IV	89 (76.1)	39 (86.7)	50 (69.4)		51 (82.3)	38 (69.1)	
Radiochemotherapy	Adjuvant	37 (31.6)	13 (28.9)	24 (33.3)	0.765	24 (38.7)	13 (23.6)	0.121
	Definitive	80 (68.4)	32 (71.1)	48 (66.7)		38 (61.3)	42 (76.4)	
Concomitant CT modification	No	75 (64.1)	26 (57.8)	49 (68.1)	0.353	39 (62.9)	36 (65.5)	0.925
	Yes	42 (35.9)	19 (42.2)	23 (31.9)		23 (37.1)	19 (34.5)	
T0 time from RT start (median [IQR])		6 [4, 12]	9 [5, 17]	6 [2, 8.25]	0.004	4 [2, 5]	12 [8.5, 20]	<0.001
T0 time from RT start (%)	<7 days	62 (53)	16 (35.6)	46 (63.9)	0.005	62 (100)	0 (0.0)	<0.001
	≥7 days	55 (47)	29 (64.4)	26 (36.1)		0 (0.0)	55 (100)	
T1_timefromRTstart (median [IQR])		24 [20, 28]	23 [18, 29]	24 [21, 28]	0.682	23 [18.25, 27]	28 [22, 32]	0.001
T2_timefromRTstart (median [IQR])		44 [42, 47]	42 [39, 44]	46 [43, 48]	<0.001	44 [41.25, 47]	44 [42, 47.5]	0.630
T3_timefromRTstart (median [IQR])		137 [135, 139]	137 [136, 139]	137 [135, 13]	0.857	136 [135, 138]	138 [136, 140.5]	0.005
T4_timefromRTstart (median [IQR])		428 [271, 544]	370 [212, 505]	453.5 [294, 568.75]	0.054	464 [315, 548.5]	383 [207, 541]	0.108
Diet (%)	normal	101 (86.3)	39 (86.7)	62 (86.1)	1.000	56 (90.3)	45 (81.8)	0.329
	oral nutritional supplements	10 (8.5)	4 (8.9)	6 (8.3)		3 (4.8)	7 (12.7)	
	invasive	6 (5.1)	2 (4.4)	4 (5.6)		3 (4.8)	3 (5.5)	
Weight Pre CRT (median [IQR])		75 [65, 88]	76 [64, 89]	74 [65.75, 84]	0.766	78.85 [65, 89]	74 [64, 80.5]	0.269
Weight End CRT (median [IQR])		70.7 [62, 80]	71.8 [63, 81]	70.35 [62, 79.25]	0.756	72 [62, 81.75]	68.4 [63, 78.2]	0.431
Weight: percentage loss (median [IQR])		3.45 [0, 7.58]	4.27 [0, 9.46]	3.32 [−0.97, 6.37]	0.320	4.08 [−1.76, 7.52]	2.88 [0, 7.62]	0.872
last_fup_weight (median [IQR])		72 [65, 82.25]	72 [66.6, 78.25]	72 [63, 84.22]	0.856	73 [64.5, 84.95]	71 [66, 75]	0.290

PBM: Photobiomodulation. CT: chemotherapy. RT: radiotherapy. T0: time at the beginning of PBM. T1: time at the mid-way point of PBM treatment. T2: time at the end of treatment. T3: time at 3 months to end treatment. T4: time at the last follow up. CRT: chemoradiotherapy. Statistically significant values are underlined.

**Table 3:** Toxicity of the overall cohort.

Toxicity	Time	Grade 0	Grade 1	Grade 2	Grade 3
Dysgeusia	T0	*	*	*	*
	T1	*	*	*	*
	T2	*	*	*	*
	T3	87 (74.4%)	18 (15.4%)	12 (10.3%)	0 (0.0%)
	T4	91 (77.8%)	22 (18.8%)	4 (3.4%)	0 (0.0%)
Dysphagia	T0	74 (63.2%)	27 (23.1%)	11 (9.4%)	5 (4.3%)
	T1	21 (17.9%)	50 (42.7%)	31 (26.5%)	15 (12.8%)
	T2	12 (10.3%)	39 (33.3%)	38 (32.5%)	28 (23.9%)
	T3	60 (51.3%)	33 (28.2%)	14 (12.0%)	10 (8.5%)
	T4	93 (79.5%)	21 (17.9%)	3 (2.6%)	0 (0.0%)
Mucositis	T0	95 (81.2%)	13 (11.1%)	6 (5.1%)	3 (2.6%)
	T1	29 (24.8%)	48 (41.0%)	33 (28.2%)	7 (6.0%)
	T2	27 (23.1%)	45 (38.5%)	39 (33.3%)	6 (5.1%)
	T3	83 (70.9%)	25 (21.4%)	8 (6.8%)	1 (0.9%)
	T4	114 (97.4%)	3 (2.6%)	0 (0.0%)	0 (0.0%)
Odynophagia	T0	103 (88.0%)	13 (11.1%)	1 (0.9%)	0 (0.0%)
	T1	66 (56.4%)	21 (17.9%)	12 (10.3%)	18 (15.4%)
	T2	45 (38.5%)	41 (35.0%)	14 (12.0%)	17 (14.5%)
	T3	88 (75.2%)	23 (19.7%)	2 (1.7%)	4 (3.4%)
	T4	117 (100%)	0 (0.0%)	0 (0.0%)	0 (0.0%)
Radiodermatitis	T0	94 (80.3%)	23 (19.7%)	0 (0.0%)	0 (0.0%)
	T1	20 (17.1%)	84 (71.8%)	11 (9.4%)	2 (1.7%)
	T2	7 (6.0%)	80 (68.4%)	27 (23.1%)	3 (2.6%)
	T3	82 (70.1%)	35 (29.9%)	0 (0.0%)	0 (0.0%)
	T4	117 (100%)	0 (0.0%)	0 (0.0%)	0 (0.0%)
Trismus	T0	110 (94.0%)	5 (4.3%)	2 (1.7%)	0 (0.0%)
	T1	106 (90.6%)	8 (6.8%)	3 (2.6%)	0 (0.0%)
	T2	101 (86.3%)	12 (10.3%)	4 (3.4%)	0 (0.0%)
	T3	107 (91.5%)	8 (6.8%)	2 (1.7%)	0 (0.0%)
	T4	107 (91.5%)	9 (7.7%)	1 (0.9%)	0 (0.0%)
Xerostomia	T0	114 (97.4%)	2 (1.7%)	1 (0.9%)	0 (0.0%)
	T1	99 (84.6%)	15 (12.8%)	3 (2.6%)	0 (0.0%)
	T2	91 (77.8%)	25 (21.4%)	1 (0.9%)	0 (0.0%)
	T3	55 (47.0%)	38 (32.5%)	24 (20.5%)	0 (0.0%)
	T4	55 (47.0%)	55 (47.0%)	7 (6.0%)	0 (0.0%)

T0: at the beginning of PBM. T1: at the mid-way point of PBM treatment. T2: at the end of treatment. T3: at 3 months to end treatment. T4: last follow up. * No evaluated during CRT/PBM as it could be influenced a lot by the ongoing chemotherapy.

**Table 4:** Association of the number of PBM sessions and timing of PBM initiation with CRT-related toxicities: logistic regression for repeated measurements (GEE, T0–T4).

Toxicity	Time Points Modeled	≥10 vs. <10 PBM Sessions OR (95% CI)	*p*-Value	Late vs. Early PBM Start OR (95% CI)	*p*-Value	Time Point (Global *p*)
A. Any grade (grade 1–3 vs. grade 0)						
Oral mucositis	T0, T1, T2, T3, T4	0.97 (0.58–1.62)	0.897	1.45 (0.87–2.40)	0.152	<0.001
Radiodermatitis	T0, T1, T2, T3	2.31 (1.25–4.27)	0.008	1.91 (1.04–3.49)	0.036	<0.001
Dysphagia	T0, T1, T2, T3, T4	0.64 (0.38–1.06)	0.084	1.72 (1.04–2.81)	0.033	<0.001
Odynophagia	T0, T1, T2, T3	0.84 (0.49–1.44)	0.533	1.23 (0.72–2.08)	0.446	<0.001
Xerostomia	T0, T1, T2, T3, T4	1.08 (0.61–1.92)	0.782	0.97 (0.56–1.70)	0.918	<0.001
Trismus	T0, T1, T2, T3, T4	0.84 (0.34–2.09)	0.706	0.65 (0.26–1.62)	0.357	0.071
Dysgeusia	T3, T4	0.94 (0.49–1.82)	0.865	0.96 (0.50–1.84)	0.891	0.516
B. Grade ≥2 (grade 2–3 vs. grade 0–1)						
Oral mucositis	T0, T1, T2, T3	1.00 (0.54–1.85)	0.998	1.55 (0.86–2.79)	0.142	<0.001
Radiodermatitis	T1, T2	2.61 (1.17–5.81)	0.019	3.30 (1.55–7.03)	0.002	0.002
Dysphagia	T0, T1, T2, T3, T4	0.78 (0.44–1.35)	0.371	1.39 (0.81–2.41)	0.232	<0.001
Odynophagia	T0, T1, T2, T3	0.70 (0.39–1.25)	0.223	1.70 (0.95–3.04)	0.072	<0.001
Xerostomia	T0, T1, T2, T3, T4	1.40 (0.65–3.00)	0.392	2.20 (1.07–4.54)	0.033	<0.001
Trismus	T0, T1, T2, T3, T4	0.85 (0.11–6.31)	0.870	0.20 (0.03–1.35)	0.099	0.180
Dysgeusia	T3, T4	1.23 (0.43–3.49)	0.702	1.48 (0.51–4.36)	0.473	0.025
C. Late toxicities (single assessment at T4, logistic regression)						
Radiodermatitis	T4	1.60 (0.46–5.59)	0.465	0.58 (0.18–1.90)	0.373	–
Radiation-induced thyroiditis	T4	0.84 (0.11–6.61)	0.866	3.35 (0.32–35.58)	0.315	–
Osteoradionecrosis	T4	0.92 (0.19–4.62)	0.923	1.51 (0.30–7.51)	0.615	–
Ototoxicity	T4	0.76 (0.27–2.14)	0.602	0.24 (0.08–0.75)	0.014	–

OR: odds ratio; CI: confidence interval; PBM: photobiomodulation; GEE: generalized estimating equations. Each row is a separate logistic GEE model (logit link, exchangeable within-patient correlation, robust standard errors) including time point (categorical, reference T0), number of PBM sessions (≥10 vs. <10; reference <10) and timing of PBM initiation (late, ≥7 days after RT start vs. early, <7 days; reference early) as predictors; each OR is adjusted for the other predictors. The time point *p*-value is a global Wald test. Time points without any event for a given toxicity/grade were not estimable and were excluded (grade ≥2 radiodermatitis at T0 and T3; grade ≥2 mucositis at T4). Radiodermatitis and odynophagia were not recorded at T4, and dysgeusia was assessed only at T3–T4. Late toxicities (panel C) were assessed once and analyzed with standard logistic regression including both PBM predictors. T0: first PBM session; T1: mid-PBM; T2: end of PBM; T3: 3 months after CRT; T4: last follow-up. Time-point ORs, mixed-model and sensitivity estimates are given in Supplementary Materials Table S3.

## Data Availability

The data presented in this study are available upon request from the corresponding author. The data are not publicly available due to privacy and ethical restrictions regarding patient confidentiality.

## Supplementary Materials

The following supporting information can be downloaded at: https://www.mdpi.com/article/10.3390/jcm15207968/s1, Table S1. Photobiomodulation Devices and Protocols by Session Number and Timing. Table S2. Toxicities According to PBM Session Count and Timing. Table S3. Full models and sensitivity analyses. Table S4. Time-specific estimates (time × PBM interaction).
