# Supplementary material for: Photobiomodulation Therapy in Head and Neck Cancer Patients Undergoing Chemoradiotherapy: Role in Toxicities Prevention
Source: J Clin Med. 2026 Oct 15;15(20):7968. doi: 10.3390/jcm15207968 (PMC13705853; doi:10.3390/jcm15207968)
Supplement: Supplementary file 1 [file jcm-15-07968-s001.zip › jcm-4517693-supplementary.pdf]

**Table S1.** Photobiomodulation Devices and Protocols by Session Number and Timing.

| | Level | Overall | <10 PBM | ≥10 PBM | P-value | Early PBM (<7 days) | Late PBM ≥7 days | P-value |
|---|---|---|---|---|---|---|---|---|
| N | | 117 | 45 | 72 | | 62 | 55 | |
| **T0_machine (%)** | ATP38 | 115 (98.3) | 44 (97.8) | 71 (98.6) | 0.623 | 62 (100.0) | 53 (96.4) | 0.219 |
| | Caremin | 1 (0.9) | 1 (2.2) | 0 (0.0) | | 0 (0.0) | 1 (1.8) | |
| | Both | 1 (0.9) | 0 (0.0) | 1 (1.4) | | 0 (0.0) | 1 (1.8) | |
| **T0_atp38_protocol (%)** | Radiodermatitis - Prevention | 54 (46.6) | 22 (50.0) | 32 (44.4) | 0.205 | 36 (58.1) | 18 (33.3) | 0.001 |
| | Radiodermatitis - Curation | 54 (46.6) | 17 (38.6) | 37 (51.4) | | 26 (41.9) | 28 (51.9) | |
| | Oral Mucositis + anti-inflammation | 8 (6.9) | 5 (11.4) | 3 (4.2) | | 0 (0.0) | 8 (14.8) | |
| **T1_machine (%)** | ATP38 | 105 (89.7) | 40 (88.9) | 65 (90.3) | 0.795 | 58 (93.5) | 47 (85.5) | 0.249 |
| | Caremin | 4 (3.4) | 1 (2.2) | 3 (4.2) | | 2 (3.2) | 2 (3.6) | |
| | Both | 8 (6.8) | 4 (8.9) | 4 (5.6) | | 2 (3.2) | 6 (10.9) | |
| **T1_atp38_protocol (%)** | Radiodermatitis - Prevention | 8 (7.2) | 6 (14.0) | 2 (2.9) | 0.074 | 4 (6.7) | 4 (7.8) | 1.000 |
| | Radiodermatitis - Curation | 70 (63.1) | 27 (62.8) | 43 (63.2) | | 38 (63.3) | 32 (62.7) | |
| | Oral Mucositis + anti-inflammation | 33 (29.7) | 10 (23.3) | 23 (33.8) | | 18 (30.0) | 15 (29.4) | |
| **T2_machine (%)** | ATP38 | 106 (90.6) | 40 (88.9) | 66 (91.7) | 0.871 | 60 (96.8) | 46 (83.6) | 0.012 |
| | Caremin | 2 (1.7) | 1 (2.2) | 1 (1.4) | | 1 (1.6) | 1 (1.8) | |
| | Both | 9 (7.7) | 4 (8.9) | 5 (6.9) | | 1 (1.6) | 8 (14.5) | |
| **T2_atp38_protocol (%)** | Radiodermatitis - Prevention | 4 (3.7) | 3 (6.8) | 1 (1.5) | 0.138 | 2 (3.4) | 2 (3.9) | 1.000 |
| | Radiodermatitis - Curation | 79 (72.5) | 28 (63.6) | 51 (78.5) | | 42 (72.4) | 37 (72.5) | |
| | Oral Mucositis + anti-inflammation | 26 (23.9) | 13 (29.5) | 13 (20.0) | | 14 (24.1) | 12 (23.5) | |

Distribution of photobiomodulation (PBM) machines (ATP38 vs Caremin) and treatment protocols at initiation (T0), midpoint (T1), and end (T2) of PBM treatment. Results are stratified by total number of PBM sessions (<10 vs ≥10) and by timing of PBM initiation (Early <7 days vs Late ≥7 days). Significant differences ($p<0.05$) are highlighted in red.

**Table S2.** Toxicities According to PBM Session Count and Timing.

| Toxicity | | | Overall | <10 PBM 10. | ≥10 PBM | p-value | Early PBM (<7 days) | Late PBM ≥7 days | p-value |
|---|---|---|---|---|---|---|---|---|---|
| | | | | | | | | | |
| | N | | 117 | 45 | 72 | NA | 62 | 55 | NA |
| T0 | Mucositis | Yes (vs. No) | 22 (18.8) | 11 (24.4) | 11 (15.3) | 0.233 | 4 (6.5) | 18 (32.7) | <0.001 |
| | Mucositis | G2+ (vs. G0-1) | 9 (7.7) | 6 (13.3) | 3 (4.2) | 0.085 | 0 (0.0) | 9 (16.4) | 0.001 |
| | Radiodermatitis | Yes (vs. No) | 23 (19.7) | 7 (15.6) | 16 (22.2) | 0.476 | 9 (14.5) | 14 (25.5) | 0.165 |
| | Radiodermatitis | G2+ (vs. G0-1) | 0 (0.0) | 0 (0.0) | 0 (0.0) | NA | 0 (0.0) | 0 (0.0) | NA |
| | Dysphagia | Yes (vs. No) | 43 (36.8) | 20 (44.4) | 23 (31.9) | 0.237 | 15 (24.2) | 28 (50.9) | 0.004 |
| | Dysphagia | G2+ (vs. G0-1) | 16 (13.7) | 8 (17.8) | 8 (11.1) | 0.408 | 5 (8.1) | 11 (20.0) | 0.104 |
| | Odynophagia | Yes (vs. No) | 14 (12.0) | 7 (15.6) | 7 (9.7) | 0.388 | 5 (8.1) | 9 (16.4) | 0.254 |
| | Odynophagia | G2+ (vs. G0-1) | 1 (0.9) | 0 (0.0) | 1 (1.4) | 1.000 | 0 (0.0) | 1 (1.8) | 0.470 |
| | Xerostomia | Yes (vs. No) | 3 (2.6) | 2 (4.4) | 1 (1.4) | 0.558 | 1 (1.6) | 2 (3.6) | 0.600 |
| | Xerostomia | G2+ (vs. G0-1) | 1 (0.9) | 0 (0.0) | 1 (1.4) | 1.000 | 0 (0.0) | 1 (1.8) | 0.470 |
| | Trismus | Yes (vs. No) | 7 (6.0) | 4 (8.9) | 3 (4.2) | 0.426 | 4 (6.5) | 3 (5.5) | 1.000 |
| | Trismus | G2+ (vs. G0-1) | 2 (1.7) | 1 (2.2) | 1 (1.4) | 1.000 | 2 (3.2) | 0 (0.0) | 0.497 |
| T1 | Mucositis | Yes (vs. No) | 88 (75.2) | 34 (75.6) | 54 (75.0) | 1.000 | 45 (72.6) | 43 (78.2) | 0.526 |
| | Mucositis | G2+ (vs. G0-1) | 40 (34.2) | 15 (33.3) | 25 (34.7) | 1.000 | 18 (29.0) | 22 (40.0) | 0.244 |
| | Radiodermatitis | Yes (vs. No) | 97 (82.9) | 33 (73.3) | 64 (88.9) | 0.043 | 50 (80.6) | 47 (85.5) | 0.624 |
| | Radiodermatitis | G2+ (vs. G0-1) | 13 (11.1) | 3 (6.7) | 10 (13.9) | 0.365 | 2 (3.2) | 11 (20.0) | 0.006 |
| | Dysphagia | Yes (vs. No) | 96 (82.1) | 39 (86.7) | 57 (79.2) | 0.335 | 48 (77.4) | 48 (87.3) | 0.228 |
| | Dysphagia | G2+ (vs. G0-1) | 46 (39.3) | 19 (42.2) | 27 (37.5) | 0.698 | 20 (32.3) | 26 (47.3) | 0.129 |
| | Odynophagia | Yes (vs. No) | 51 (43.6) | 20 (44.4) | 31 (43.1) | 1.000 | 25 (40.3) | 26 (47.3) | 0.462 |
| | Odynophagia | G2+ (vs. G0-1) | 30 (25.6) | 12 (26.7) | 18 (25.0) | 0.832 | 11 (17.7) | 19 (34.5) | 0.055 |
| | Xerostomia | Yes (vs. No) | 18 (15.4) | 8 (17.8) | 10 (13.9) | 0.605 | 9 (14.5) | 9 (16.4) | 0.803 |
| | Xerostomia | G2+ (vs. G0-1) | 3 (2.6) | 2 (4.4) | 1 (1.4) | 0.558 | 0 (0.0) | 3 (5.5) | 0.101 |
| | Trismus | Yes (vs. No) | 11 (9.4) | 4 (8.9) | 7 (9.7) | 1.000 | 6 (9.7) | 5 (9.1) | 1.000 |
| | Trismus | G2+ (vs. G0-1) | 3 (2.6) | 1 (2.2) | 2 (2.8) | 1.000 | 2 (3.2) | 1 (1.8) | 1.000 |
| T2 | Mucositis | Yes (vs. No) | 90 (76.9) | 37 (82.2) | 53 (73.6) | 0.368 | 47 (75.8) | 43 (78.2) | 0.828 |
| | Mucositis | G2+ (vs. G0-1) | 45 (38.5) | 19 (42.2) | 26 (36.1) | 0.561 | 22 (35.5) | 23 (41.8) | 0.569 |
| | Radiodermatitis | Yes (vs. No) | 110 (94.0) | 41 (91.1) | 69 (95.8) | 0.426 | 59 (95.2) | 51 (92.7) | 0.705 |
| | Radiodermatitis | G2+ (vs. G0-1) | 30 (25.6) | 9 (20.0) | 21 (29.2) | 0.288 | 13 (21.0) | 17 (30.9) | 0.289 |
| | Dysphagia | Yes (vs. No) | 105 (89.7) | 40 (88.9) | 65 (90.3) | 1.000 | 56 (90.3) | 49 (89.1) | 1.000 |
| | Dysphagia | G2+ (vs. G0-1) | 66 (56.4) | 29 (64.4) | 37 (51.4) | 0.184 | 34 (54.8) | 32 (58.2) | 0.852 |
| | Odynophagia | Yes (vs. No) | 72 (61.5) | 31 (68.9) | 41 (56.9) | 0.243 | 35 (56.5) | 37 (67.3) | 0.257 |

| | | | | | | | | | |
|---|---|---|---|---|---|---|---|---|---|
| | Odynophagia | G2+ (vs. G0-1) | 31 (26.5) | 19 (42.2) | 12 (16.7) | 0.005 | 13 (21.0) | 18 (32.7) | 0.208 |
| | Xerostomia | Yes (vs. No) | 26 (22.2) | 9 (20.0) | 17 (23.6) | 0.820 | 14 (22.6) | 12 (21.8) | 1.000 |
| | Xerostomia | G2+ (vs. G0-1) | 1 (0.9) | 0 (0.0) | 1 (1.4) | 1.000 | 0 (0.0) | 1 (1.8) | 0.470 |
| | Trismus | Yes (vs. No) | 16 (13.7) | 6 (13.3) | 10 (13.9) | 1.000 | 10 (16.1) | 6 (10.9) | 0.436 |
| | Trismus | G2+ (vs. G0-1) | 4 (3.4) | 1 (2.2) | 3 (4.2) | 1.000 | 4 (6.5) | 0 (0.0) | 0.121 |
| **T3** | Mucositis | Yes (vs. No) | 34 (29.1) | 11 (24.4) | 23 (31.9) | 0.411 | 20 (32.3) | 14 (25.5) | 0.541 |
| | Mucositis | G2+ (vs. G0-1) | 9 (7.7) | 2 (4.4) | 7 (9.7) | 0.479 | 6 (9.7) | 3 (5.5) | 0.498 |
| | Radiodermatitis | Yes (vs. No) | 35 (29.9) | 11 (24.4) | 24 (33.3) | 0.407 | 16 (25.8) | 19 (34.5) | 0.320 |
| | Radiodermatitis | G2+ (vs. G0-1) | 0 (0.0) | 0 (0.0) | 0 (0.0) | NA | 0 (0.0) | 0 (0.0) | NA |
| | Dysphagia | Yes (vs. No) | 57 (48.7) | 25 (55.6) | 32 (44.4) | 0.260 | 29 (46.8) | 28 (50.9) | 0.713 |
| | Dysphagia | G2+ (vs. G0-1) | 24 (20.5) | 10 (22.2) | 14 (19.4) | 0.815 | 12 (19.4) | 12 (21.8) | 0.820 |
| | Dysgeusia | Yes (vs. No) | 30 (25.6) | 10 (22.2) | 20 (27.8) | 0.664 | 16 (25.8) | 14 (25.5) | 1.000 |
| | Dysgeusia | G2+ (vs. G0-1) | 12 (10.3) | 4 (8.9) | 8 (11.1) | 0.765 | 6 (9.7) | 6 (10.9) | 1.000 |
| | Odynophagia | Yes (vs. No) | 29 (24.8) | 11 (24.4) | 18 (25.0) | 1.000 | 17 (27.4) | 12 (21.8) | 0.526 |
| | Odynophagia | G2+ (vs. G0-1) | 6 (5.1) | 1 (2.2) | 5 (6.9) | 0.404 | 4 (6.5) | 2 (3.6) | 0.683 |
| | Xerostomia | Yes (vs. No) | 62 (53.0) | 21 (46.7) | 41 (56.9) | 0.342 | 33 (53.2) | 29 (52.7) | 1.000 |
| | Xerostomia | G2+ (vs. G0-1) | 24 (20.5) | 6 (13.3) | 18 (25.0) | 0.161 | 12 (19.4) | 12 (21.8) | 0.820 |
| | Trismus | Yes (vs. No) | 10 (8.5) | 4 (8.9) | 6 (8.3) | 1.000 | 7 (11.3) | 3 (5.5) | 0.331 |
| | Trismus | G2+ (vs. G0-1) | 2 (1.7) | 1 (2.2) | 1 (1.4) | 1.000 | 1 (1.6) | 1 (1.8) | 1.000 |
| **T4** | Xerostomia | Yes (vs. No) | 62 (53.0) | 25 (55.6) | 37 (51.4) | 0.706 | 34 (54.8) | 28 (50.9) | 0.713 |
| | Xerostomia | G2+ (vs. G0-1) | 7 (6.0) | 5 (11.1) | 2 (2.8) | 0.105 | 2 (3.2) | 5 (9.1) | 0.251 |
| | Dysphagia | Yes (vs. No) | 24 (20.5) | 15 (33.3) | 9 (12.5) | 0.009 | 8 (12.9) | 16 (29.1) | 0.039 |
| | Dysphagia | G2+ (vs. G0-1) | 3 (2.6) | 2 (4.4) | 1 (1.4) | 0.558 | 0 (0.0) | 3 (5.5) | 0.101 |
| | Dysgeusia | Yes (vs. No) | 26 (22.2) | 12 (26.7) | 14 (19.4) | 0.371 | 14 (22.6) | 12 (21.8) | 1.000 |
| | Dysgeusia | G2+ (vs. G0-1) | 4 (3.4) | 2 (4.4) | 2 (2.8) | 0.638 | 1 (1.6) | 3 (5.5) | 0.341 |
| | Trismus | Yes (vs. No) | 10 (8.5) | 4 (8.9) | 6 (8.3) | 1.000 | 6 (9.7) | 4 (7.3) | 0.748 |
| | Trismus | G2+ (vs. G0-1) | 1 (0.9) | 0 (0.0) | 1 (1.4) | 1.000 | 1 (1.6) | 0 (0.0) | 1.000 |
| | Mucositis | Yes (vs. No) | 3 (2.6) | 1 (2.2) | 2 (2.8) | 1.000 | 1 (1.6) | 2 (3.6) | 0.600 |
| | Mucositis | G2+ (vs. G0-1) | 0 (0.0) | 0 (0.0) | 0 (0.0) | NA | 0 (0.0) | 0 (0.0) | NA |
| | Radiodermatitis | Yes (vs. No) | 15 (12.8) | 4 (8.9) | 11 (15.3) | 0.401 | 10 (16.1) | 5 (9.1) | 0.390 |
| | Radiation induced thyroiditis | Yes (vs. No) | 4 (3.4) | 2 (4.4) | 2 (2.8) | 0.638 | 1 (1.6) | 3 (5.5) | 0.341 |
| | Osteoradionecrosis | Yes (vs. No) | 7 (6.0) | 3 (6.7) | 4 (5.6) | 1.000 | 3 (4.8) | 4 (7.3) | 0.705 |
| | Ototoxicity | Yes (vs. No) | 22 (18.8) | 8 (17.8) | 14 (19.4) | 1.000 | 17 (27.4) | 5 (9.1) | 0.022 |

PBM: Photobiomodulation. T0: time at the beginning of PBM. T1: time at half of PBM treatment. T2: time at end of treatment. T3: time at 3 months to end treatment. T4: time last follow up.

**Table S3 – Full models and sensitivity analyses**

| Toxicity | Predictor | GEE (primary) OR (95% CI) | p | Mixed model OR (95% CI) | p | GEE excluding T0 OR (95% CI) | p |
|---|---|---|---|---|---|---|---|
| **A. Any grade (grade 1–3 vs 0)** | | | | | | | |
| Oral mucositis | Time: T1 vs T0 | 13.38 (7.73–23.16) | <0.001 | 19.34 (9.21–40.62) | <0.001 | – | – |
| | Time: T2 vs T0 | 14.71 (8.27–26.17) | <0.001 | 21.51 (10.13–45.71) | <0.001 | – | – |
| | Time: T3 vs T0 | 1.78 (0.93–3.39) | 0.082 | 1.90 (0.99–3.66) | 0.053 | – | – |
| | Time: T4 vs T0 | 0.11 (0.03–0.40) | <0.001 | 0.10 (0.03–0.35) | <0.001 | – | – |
| | ≥10 vs <10 PBM sessions | 0.97 (0.58–1.62) | 0.897 | 0.97 (0.54–1.73) | 0.916 | 0.99 (0.56–1.74) | 0.967 |
| | Late (≥7 d) vs early PBM start | 1.45 (0.87–2.40) | 0.152 | 1.55 (0.88–2.73) | 0.133 | 1.03 (0.59–1.80) | 0.912 |
| Radiodermatitis | Time: T1 vs T0 | 22.35 (12.30–40.62) | <0.001 | 36.13 (14.86–87.87) | <0.001 | – | – |
| | Time: T2 vs T0 | 74.25 (30.14–182.94) | <0.001 | 134.56 (42.55–425.51) | <0.001 | – | – |
| | Time: T3 vs T0 | 1.78 (1.00–3.16) | 0.051 | 1.93 (1.00–3.74) | 0.052 | – | – |
| | ≥10 vs <10 PBM sessions | 2.31 (1.25–4.27) | 0.008 | 2.57 (1.29–5.08) | 0.007 | 2.39 (1.24–4.59) | 0.009 |
| | Late (≥7 d) vs early PBM start | 1.91 (1.04–3.49) | 0.036 | 2.03 (1.05–3.91) | 0.035 | 1.69 (0.88–3.23) | 0.114 |
| Dysphagia | Time: T1 vs T0 | 8.42 (4.95–14.30) | <0.001 | 11.84 (5.87–23.89) | <0.001 | – | – |
| | Time: T2 vs T0 | 16.32 (8.43–31.58) | <0.001 | 24.59 (10.89–55.53) | <0.001 | – | – |
| | Time: T3 vs T0 | 1.66 (1.02–2.71) | 0.041 | 1.82 (1.02–3.25) | 0.043 | – | – |
| | Time: T4 vs T0 | 0.43 (0.24–0.78) | 0.005 | 0.38 (0.20–0.72) | 0.003 | – | – |
| | ≥10 vs <10 PBM sessions | 0.64 (0.38–1.06) | 0.084 | 0.60 (0.34–1.08) | 0.087 | 0.59 (0.34–1.02) | 0.059 |

| | | | | | | | |
|---|---|---|---|---|---|---|---|
| | Late (≥7 d) vs early PBM start | 1.72 (1.04–2.81) | 0.033 | 1.83 (1.04–3.22) | 0.036 | 1.37 (0.82–2.31) | 0.231 |
| Odynophagia | Time: T1 vs T0 | 5.73 (3.20–10.26) | <0.001 | 8.40 (3.89–18.11) | <0.001 | – | – |
| | Time: T2 vs T0 | 11.91 (6.24–22.73) | <0.001 | 21.40 (9.40–48.68) | <0.001 | – | – |
| | Time: T3 vs T0 | 2.43 (1.20–4.95) | 0.014 | 2.87 (1.33–6.18) | 0.007 | – | – |
| | ≥10 vs <10 PBM sessions | 0.84 (0.49–1.44) | 0.533 | 0.80 (0.41–1.57) | 0.515 | 0.86 (0.49–1.51) | 0.598 |
| | Late (≥7 d) vs early PBM start | 1.23 (0.72–2.08) | 0.446 | 1.30 (0.67–2.52) | 0.435 | 1.17 (0.67–2.04) | 0.588 |
| Xerostomia | Time: T1 vs T0 | 6.91 (2.21–21.66) | <0.001 | 8.43 (2.29–31.08) | 0.001 | – | – |
| | Time: T2 vs T0 | 10.87 (3.42–34.49) | <0.001 | 14.44 (3.99–52.27) | <0.001 | – | – |
| | Time: T3 vs T0 | 42.89 (12.58–146.25) | <0.001 | 79.75 (21.55–295.20) | <0.001 | – | – |
| | Time: T4 vs T0 | 42.89 (12.13–151.67) | <0.001 | 79.75 (21.55–295.20) | <0.001 | – | – |
| | ≥10 vs <10 PBM sessions | 1.08 (0.61–1.92) | 0.782 | 1.04 (0.54–2.01) | 0.896 | 1.08 (0.61–1.91) | 0.783 |
| | Late (≥7 d) vs early PBM start | 0.97 (0.56–1.70) | 0.918 | 1.00 (0.53–1.89) | 0.996 | 0.97 (0.56–1.69) | 0.917 |
| Trismus | Time: T1 vs T0 | 1.63 (0.83–3.22) | 0.157 | 2.01 (0.62–6.54) | 0.246 | – | – |
| | Time: T2 vs T0 | 2.50 (1.22–5.13) | 0.013 | 3.82 (1.23–11.91) | 0.021 | – | – |
| | Time: T3 vs T0 | 1.47 (0.59–3.65) | 0.406 | 1.73 (0.52–5.70) | 0.371 | – | – |
| | Time: T4 vs T0 | 1.47 (0.55–3.91) | 0.440 | 1.73 (0.52–5.70) | 0.371 | – | – |
| | ≥10 vs <10 PBM sessions | 0.84 (0.34–2.09) | 0.706 | 0.63 (0.17–2.34) | 0.487 | 0.90 (0.36–2.21) | 0.813 |
| | Late (≥7 d) vs early PBM start | 0.65 (0.26–1.62) | 0.357 | 0.57 (0.16–2.10) | 0.400 | 0.65 (0.26–1.64) | 0.364 |
| Dysgeusia | Time: T4 vs T0 | 0.83 (0.47–1.46) | 0.516 | 0.81 (0.43–1.53) | 0.517 | – | – |

| | | | | | | | |
|---|---|---|---|---|---|---|---|
| | ≥10 vs <10 PBM sessions | 0.94 (0.49–1.82) | 0.865 | 0.93 (0.43–1.99) | 0.851 | 0.94 (0.49–1.82) | 0.865 |
| | Late (≥7 d) vs early PBM start | 0.96 (0.50–1.84) | 0.891 | 0.95 (0.45–2.00) | 0.897 | 0.96 (0.50–1.84) | 0.891 |
| **B. Grade ≥2 (grade 2–3 vs 0–1)** | | | | | | | |
| Oral mucositis | Time: T1 vs T0 | 6.31 (3.12–12.76) | <0.001 | 11.06 (4.30–28.46) | <0.001 | – | – |
| | Time: T2 vs T0 | 7.61 (3.75–15.47) | <0.001 | 14.34 (5.51–37.35) | <0.001 | – | – |
| | Time: T3 vs T0 | 1.00 (0.39–2.57) | 1.000 | 1.00 (0.35–2.88) | 1.000 | – | – |
| | ≥10 vs <10 PBM sessions | 1.00 (0.54–1.85) | 0.998 | 1.04 (0.45–2.42) | 0.921 | 1.04 (0.55–1.94) | 0.907 |
| | Late (≥7 d) vs early PBM start | 1.55 (0.86–2.79) | 0.142 | 1.97 (0.86–4.49) | 0.109 | 1.40 (0.76–2.57) | 0.282 |
| Radiodermatitis | Time: T2 vs T0 | 2.93 (1.50–5.76) | 0.002 | 3.43 (1.48–7.96) | 0.004 | – | – |
| | ≥10 vs <10 PBM sessions | 2.61 (1.17–5.81) | 0.019 | 3.10 (1.14–8.45) | 0.027 | 2.61 (1.17–5.81) | 0.019 |
| | Late (≥7 d) vs early PBM start | 3.30 (1.55–7.03) | 0.002 | 4.09 (1.54–10.87) | 0.005 | 3.30 (1.55–7.03) | 0.002 |
| Dysphagia | Time: T1 vs T0 | 4.15 (2.45–7.05) | <0.001 | 5.93 (2.80–12.56) | <0.001 | – | – |
| | Time: T2 vs T0 | 8.38 (4.72–14.90) | <0.001 | 14.66 (6.69–32.13) | <0.001 | – | – |
| | Time: T3 vs T0 | 1.64 (0.91–2.94) | 0.100 | 1.82 (0.84–3.94) | 0.127 | – | – |
| | Time: T4 vs T0 | 0.17 (0.05–0.56) | 0.004 | 0.13 (0.03–0.50) | 0.003 | – | – |
| | ≥10 vs <10 PBM sessions | 0.78 (0.44–1.35) | 0.371 | 0.72 (0.37–1.43) | 0.352 | 0.78 (0.45–1.33) | 0.354 |
| | Late (≥7 d) vs early PBM start | 1.39 (0.81–2.41) | 0.232 | 1.67 (0.85–3.26) | 0.135 | 1.32 (0.78–2.23) | 0.303 |
| Odynophagia | Time: T1 vs T0 | 41.45 (5.83–294.53) | <0.001 | 48.04 (6.19–372.55) | <0.001 | – | – |
| | Time: T2 vs T0 | 43.38 (5.58–336.92) | <0.001 | 50.49 (6.51–391.69) | <0.001 | – | – |
| | Time: T3 vs T0 | 6.32 (0.73–54.96) | 0.095 | 6.51 (0.76–55.76) | 0.087 | – | – |

| | | | | | | | |
|---|---|---|---|---|---|---|---|
| | ≥10 vs <10 PBM sessions | 0.70 (0.39–1.25) | 0.223 | 0.71 (0.36–1.40) | 0.326 | 0.67 (0.37–1.20) | 0.178 |
| | Late (≥7 d) vs early PBM start | 1.70 (0.95–3.04) | 0.072 | 1.79 (0.91–3.49) | 0.089 | 1.68 (0.94–2.99) | 0.081 |
| Xerostomia | Time: T1 vs T0 | 3.06 (0.31–30.49) | 0.340 | 3.06 (0.31–29.91) | 0.336 | – | – |
| | Time: T2 vs T0 | 1.00 (0.06–16.47) | 1.000 | 1.00 (0.06–16.21) | 1.000 | – | – |
| | Time: T3 vs T0 | 30.82 (4.00–237.18) | <0.001 | 30.76 (4.07–232.34) | <0.001 | – | – |
| | Time: T4 vs T0 | 7.44 (0.88–62.78) | 0.065 | 7.43 (0.90–61.55) | 0.063 | – | – |
| | ≥10 vs <10 PBM sessions | 1.40 (0.65–3.00) | 0.392 | 1.39 (0.65–3.01) | 0.397 | 1.30 (0.59–2.83) | 0.515 |
| | Late (≥7 d) vs early PBM start | 2.20 (1.07–4.54) | 0.033 | 2.14 (1.01–4.56) | 0.048 | 2.01 (0.95–4.22) | 0.066 |
| Trismus | Time: T1 vs T0 | 1.52 (0.67–3.43) | 0.315 | 1.86 (0.20–17.05) | 0.583 | – | – |
| | Time: T2 vs T0 | 2.05 (0.75–5.58) | 0.160 | 2.98 (0.35–25.45) | 0.318 | – | – |
| | Time: T3 vs T0 | 1.00 (0.13–7.45) | 1.000 | 1.00 (0.09–10.75) | 1.000 | – | – |
| | Time: T4 vs T0 | 0.49 (0.04–5.68) | 0.571 | 0.39 (0.02–6.37) | 0.505 | – | – |
| | ≥10 vs <10 PBM sessions | 0.85 (0.11–6.31) | 0.870 | 1.17 (0.08–17.64) | 0.911 | 1.04 (0.16–6.64) | 0.965 |
| | Late (≥7 d) vs early PBM start | 0.20 (0.03–1.35) | 0.099 | 0.23 (0.01–4.19) | 0.320 | 0.27 (0.05–1.61) | 0.151 |
| Dysgeusia | Time: T4 vs T0 | 0.31 (0.11–0.86) | 0.025 | 0.20 (0.04–0.90) | 0.036 | – | – |
| | ≥10 vs <10 PBM sessions | 1.23 (0.43–3.49) | 0.702 | 1.22 (0.21–6.93) | 0.826 | 1.23 (0.43–3.49) | 0.702 |
| | Late (≥7 d) vs early PBM start | 1.48 (0.51–4.36) | 0.473 | 1.95 (0.35–10.99) | 0.450 | 1.48 (0.51–4.36) | 0.473 |

*GEE: logistic generalized estimating equations, exchangeable correlation, robust SE (population-averaged OR). Mixed model: logistic generalized linear mixed model with a random intercept per patient (adaptive Gauss–Hermite quadrature, 10 points; subject-specific OR). Excluding T0: GEE refitted on T1–T4 only (time ORs not shown, as the reference changes). Time point reference: T0 (T3 for dysgeusia; T1 for grade ≥2 radiodermatitis). Wide time-point CIs reflect very few events at T0. Non-estimable time points (no events) were excluded (see Table 4 footnote).*

Benjamini–Hochberg q-values across the 28 PBM tests: lowest q = 0.055 (late start, grade ≥2 radiodermatitis); all others ≥ 0.11 (see Table4_GEE_main.csv).

**Table S4 – Time-specific estimates (time × PBM interaction)**

| Outcome | Time point | ≥10 vs <10 sessions OR (95% CI) | p | Late vs early start OR (95% CI) | p |
|---|---|---|---|---|---|
| **Grade ≥2 odynophagia (T1–T3) – interaction p: sessions 0.026, timing 0.295** | | | | | |
| | T1 | 1.21 (0.52–2.83) | 0.655 | 2.59 (1.11–6.06) | 0.028 |
| | T2 | 0.29 (0.13–0.68) | 0.005 | 1.31 (0.56–3.07) | 0.530 |
| | T3 | 2.91 (0.40–21.05) | 0.291 | 0.74 (0.15–3.68) | 0.718 |
| **Any-grade dysphagia (T0–T4) – interaction p: sessions 0.463, timing 0.122** | | | | | |
| | T0 | 0.79 (0.34–1.84) | 0.584 | 3.07 (1.34–7.01) | 0.008 |
| | T1 | 0.69 (0.23–2.02) | 0.495 | 1.83 (0.65–5.13) | 0.249 |
| | T2 | 1.13 (0.31–4.14) | 0.849 | 0.91 (0.25–3.28) | 0.884 |
| | T3 | 0.65 (0.30–1.42) | 0.282 | 1.06 (0.49–2.26) | 0.888 |
| | T4 | 0.35 (0.14–0.89) | 0.028 | 2.13 (0.81–5.62) | 0.127 |

*GEE model including time × sessions and time × timing interactions. Grade ≥2 odynophagia: T0 (1 event) and T4 (no event) excluded. Interaction p: global Wald test of the time × predictor terms.*
